# Urban built environment configuration and psychological distress in older men: Results from the Caerphilly study

**DOI:** 10.1186/1471-2458-13-695

**Published:** 2013-07-30

**Authors:** Chinmoy Sarkar, John Gallacher, Chris Webster

**Affiliations:** 1 School of Planning and Geography, Glamorgan Building, Cardiff University, Cardiff CF10 3WA, UK; 2 Department of Primary Care and Public Health, Centre for Health Sciences Research, School of Medicine, Cardiff University, Cardiff CF14 4XN, UK; 3 Faculty of Architecture, The University of Hong Kong, Hong Kong, China

**Keywords:** Psychological distress, GHQ, Older adults, Built environment configuration, Space syntax, Random effects, Runmlwin

## Abstract

**Background:**

Few studies have examined the impact of the built environment configuration upon mental health. The study examines the impact of objectively assessed land use and street network configuration upon psychological distress and whether this association is moderated by the natural environment and area-level deprivation.

**Methods:**

In a community sample of 687 older men from the Caerphilly Prospective Study, built environment morphological metrics (morphometrics) were related to differences in psychological distress as measured by the General Health Questionnaire. Cross-sectional data were taken from the most recent (5th) phase. A multi-level analysis with individuals nested within census-defined neighbourhoods was conducted. Environmental measures comprised GIS-constructed land use and street network metrics, slope variability and a satellite derived measure of greenness.

**Results:**

Reduced psychological distress was associated with residing in a terraced dwelling (OR = 0.48, p = 0.03), higher degrees of land-use mix (OR = 0.42, p = 0.03 for the high tertile) and having higher local-level street-network accessibility (‘movement potential’) (OR = 0.54, p = 0.03). Hillier topography with higher slope variability was associated with increased risks of psychological distress (OR = 1.38, p = 0.05).

**Conclusions:**

The findings support our hypothesis that built environment configuration is independently associated with psychological distress. The study underscores the need for effective intervention in the planning and design of residential built environment to achieve the goal of health-sustaining communities.

## Background

There is increasing evidence that the built environment affects mental health. Associations have been found between psychological wellbeing and housing structural attributes [1-3], dwelling level architectural features [4,5], observable neighbourhood environmental quality [6,7], access to facilities and green space [8-10], and neighbourhood walkability [11,12]. Most studies have either used composite [6,7,11,12] or self-report [10,13] measures of the built environment. A recent study reported a positive association between low psychosocial distress and residing in neighbourhoods with higher quality of public open space [14]. However, only one study has examined the association between objectively measured components of the built environment and mental health in older adults, finding higher levels of depression with higher degrees of land-use mix and greater retail availability [15]. In this paper we investigate, in greater detail, the relationship between objectively measured morphological characteristics of the built environment and mental health.

Objective assessment of the built environment is a fast growing area. Following Lynch, who conceptualised five elements used by individuals in the construction of cognitive maps for navigating through urban space, in the form of nodes (strategic areas), paths, edges, districts, and landmarks [16], various topological schema have been suggested [17]. More recently, Hillier has attempted to quantify the relationship between urban spatial configuration and social processes [18-20]. Hillier proposed that urban space comprises a set of discrete interconnected units (sub-spaces) classified in to three typologies: linear space in the form of street segments through which people move, convex space comprising of squares and public spaces through which they interact, and the ever changing visual field through which they perceive their surroundings. It is primarily the boundaries, physical connectivity and accessibility of these sub-spaces that govern mental and behavioural and social responses.

In the present study we hypothesize that urban configuration; both land use (mix, density, and destination accessibility) as well as street networks (connectivity and movement potential) enhances social cohesion by encouraging social interaction and improving accessibility to health promoting community resources, resulting in mental health benefits (whilst acknowledging the presence of certain destinations that may serve to inhibit social cohesion). The mental health benefits of neighbourhood walkability and physical activity upon psychological disorder have been established [12,21-24], as have the effects of social cohesion [25-27]. We also suggest these relationships are of particular importance in older adults, whose daily activities tend usually to be confined to the vicinity of their dwelling, making them more vulnerable to the health promoting/inhibiting attributes of neighbourhood physical configuration [28,29]. We test our hypothesis by examining the association of a wide range of morphological metrics (morphometrics) with psychological distress in a population of older men after adjusting for natural environment, social deprivation and individual level factors.

## Methods

### Study design

The study is based on the 5th phase of the Caerphilly Prospective Study (CaPS), a population-based male cohort in the Welsh Assembly constituency of Caerphilly, South Wales, UK (83,600 inhabitants over 114.54 sq km with a density of 727 inhabitants/km^2^). Further details of the CaPS cohort can be found elsewhere [[30], http://www.bris.ac.uk/social-community-medicine/projects/caerphilly/about/]. Briefly, the 5th phase was conducted during 2002–2004 and comprised 1225 surviving cohort members aged 65–84 years. Extensive clinical examinations including anthropometry, assessment of blood pressure, electrocardiogram as well as collection of fasting blood samples. The research protocol was approved by the South Wales research ethics committee and written informed consent was obtained from all participants.

### Psychological distress

Psychological distress was measured using the 30-item General Health Questionnaire (GHQ-30), a self-completion instrument widely used to rate levels of psychological distress and psychiatric disorders [31].

### Individual level covariates

Individual level covariates comprised age, alcohol consumption, social class and educational attainment. The study also controlled for the existence of chronic vascular morbidities, expressed in terms of prevalence of any one or more of myocardial infarction, angina, high blood pressure, high cholesterol, diabetes and stroke.

### The built environment

Built environment measures were operationalised within a 1-kilometre street-network buffer around an individual respondent’s dwelling unit. Measures were derived from the three layers of UK Ordnance Survey Master Map (OSMM) data set; namely, the Topography Layer, Integrated Transport Network Layer (ITN) and Address Layer 2. Geocoding was successful for 94% of the respondent’s residences. The OSMM Topographic Layer and Address Layer 2 were cleaned and buildings and dwellings tables extracted. These tables provided the information on dwellings per building, Royal Mail address and the OSMM land use classes within each building unit. The OSMM ITN layer provides a topologically structured representation of the road network, containing information about link length, geometry, junctions, names etc. It was subjected to network analysis in propriety space syntax software, Confeego [32]. From these were constructed 13 measures of built environment morphological metrics under three categories, namely dwelling level, land use and street-network accessibility variables. Spatial data analysis, integration and compilation were performed within ArcGIS 9.3.

Dwelling-level configurations included measures of plot exposure, dwelling-centered density and dwelling type. Plot exposure evaluated the number of faces of a dwelling unit exposed to public space and was categorized as zero, one and more than one, with one acting as the reference category. Dwelling-centred density was measured as the number of dwelling units within a 30m kernel surrounding the dwelling. Dwelling type was categorized as: detached, semi-detached, terraced and flats, with detached acting as the reference category.

Land use configurations were captured through measures of mix and density. A five-category land-use mix score was calculated using the method proposed by Frank et al. [33]. This measured land area under residential dwellings, retail, community services, business and offices, and recreation and leisure, calculated as:

$$\mathrm{LUM}=\left[\frac{‒\sum_{k=1}^{n}\left(p_{k}\cdot \ln\;p_{k}\right)}{\ln\;N}\right]$$

where p is the proportion of land area devoted to specific land use category k and N is the number of land uses. The land use mix index ranges between 0–1, with 0 representing a homogeneous, single land use environment and 1 representing a perfectly heterogeneous neighbourhood.

In addition, the densities of public transit (bus stops), retail, community services, recreation and leisure, and business and offices were calculated as the number of units divided by the total area within a 1 kilometre street-network buffer of a respondents dwelling unit.

**Figure 1:**
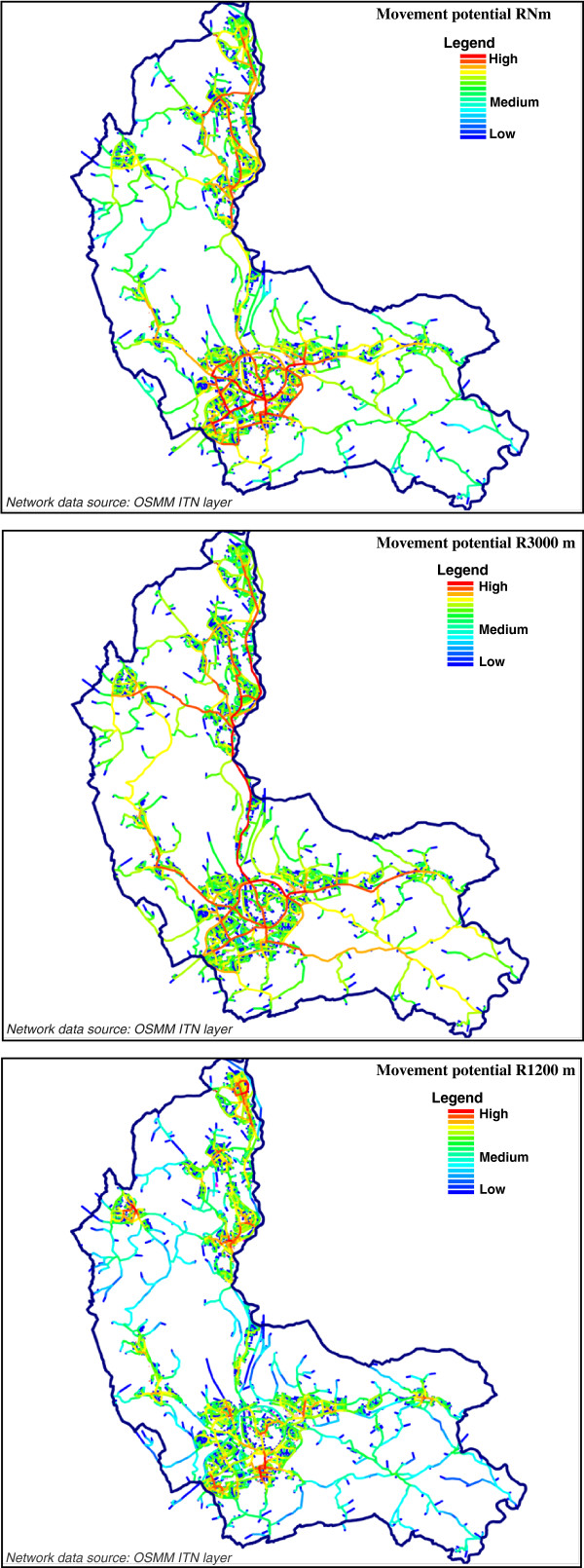
**Analysis of street movement potential at various scales for the study area.** Space syntax based network analysis was performed over the Ordnance Survey ITN layer. © Crown copyright UK Ordnance Survey. All rights reserved.

Space syntax modeling [18-20,34,35] measures the physical accessibility of street network segments and has been previously employed in health research [36]. We employ this modelling technique in the present study to assess the impacts of street-level accessibility on mental health via their influences upon physical activity and social capital. The OSMM ITN layer of Caerphilly was edited, simplified and transcribed in to a road centreline map, which is essentially a model of the street network configuration using the simplest set of line segments representing the longest lines of visual sight. This essentially enables us to capture the effects of changes in direction and the presence of intervening streets upon individual’s sense of orientation in an urban space. Angular segment analysis [37] of the street-network was performed on the OSMM ITN layer in Confeego to measure physical accessibility of street segments. Two indices of physical accessibility –*street movement potential* (also known in network analysis terminology as *betweenness*) and *connectivity* were employed. They assess the degree of *connectedness* as well as *walkability* of each street-network segment in an urban network; thereby, acting as proxies for *accessible neighbourhood* promoting *active living*. *Street movement potential* measures the amount of passing movements through a street segment and is synonymous with the propensity of a street segment being used for a particular trip. It can be measured at multiple spatial scales. In our models, the movement potential was measured for each street segment with reference to networks defined at three spatial scales (Figure 1). At a local scale, movement potential was measured at a 1200-metre network catchment (equivalent to a 15 minute stroll), with higher values being synonymous with higher street-level walkability and hence greater pedestrian activity, especially in terms of walking for transport, shopping and recreation. Given the spatial extent of Caerphilly a radius of 3000 metres was employed to measure city-scale accessibility. Movement potential at this scale represents city-scale accessibility to various service utilities and captures degree of public transport and car-usage. At N meters (comprising all network segments in the system), movement-potential becomes an index of regional accessibility capturing long distance transit behaviour. Higher regional-level movement-potential such as being located near motorways generally creates an environment that is detrimental to walking trips and physical activity, being synonymous with traffic density, congestion and pollution.

Street *connectivity* measures the number of segments connected to a segment and is synonymous with the density of street intersections. For older adults, a higher intersection density may inhibit active living on account of higher traffic density and the resulting lack of road safety while crossing streets.

### Natural environment

Natural environmental measures of slope variability and greenness were included in the models, the former being particularly variable given the geographical location in the Welsh Valleys. Slope raster was processed from a 5-metre resolution Blue Sky digital terrain model of the study area and the degree of variability in slope within an individual’s home range was operationalized as the standard deviation of slope in degrees within a 1 kilometer network buffer of an individual’s dwelling. Satellite derived Normalized Difference Vegetation Index (NDVI) was used as an objective measure of greenness. A 30-metre resolution Landsat 7 dataset was processed and greenness was calculated as the mean NDVI within a 500 metre circular buffer of an individual’s dwelling.

### Area-level deprivation

In order to study the effect of area-level deprivation upon psychological distress, Welsh index of multiple deprivation (WIMD) scores were measured at the level of Lower Super Output Area (LSOA). Lower super output areas are small area geographies defined by UK Office of National Statistics with a reasonable degrees of homogeneity in shape and social composition and with an average population of 1500 persons for Wales [38,39]. The LSOAs have been considered as level 2 neighbourhoods in our statistical analyses. The WIMD comprises seven unitless indicators of disadvantage (so-called *domain indices*) for income, employment, health, education, housing, access to services and physical environment [40]. In the present analysis the domain ‘access to services’ domain was not included due to the potential for collinearity.

### Statistical methods

The prevalence of psychological distress was modeled as a two-level factor (case vs. non-case) using the standard cut-point (≥5) which was confirmed by a prior validation study in this cohort [41].

Land use mix was transformed into tertiles (low, medium, high) while standardized z-scores were used for topological accessibility variables to enhance the interpretation of results. For area level variables, deprivation domain scores ranged between 0–100 with higher scores indicating more deprivation and slope variability and NDVI modeled as standardized z scores.

The individual level covariates of age (years) and alcohol consumption (ml/week) were modeled as continuous variables. Social class was expressed in terms of six groups following the Registrar General’s occupational classification [42] and thereafter collapsed into a three-level factor (I, II, III-non-manual/III-manual/IV, V). Education was modelled as a three level factor (none/apprenticeship, school certificate/higher technical certificate, professional qualification/degree/higher degree). Prevalence of chronic vascular morbidities was modelled as a two level factor, expressed in terms of presence/absence of one or more of the chronic conditions of myocardial infarction, angina, high blood pressure, high cholesterol, diabetes, and stroke. Apart from age, which was included as a standard covariate, individual covariates were included in the models if there was some evidence of association with mental health.

The impact of built environment configuration upon psychological distress was examined through a multi-level logistic regression model with individual respondents being nested within census-defined LSOAs. Two-level logistic mixed effects models with LSOA-level random effects were fitted on the case/non-case GHQ scores. The analytic plan consisted of three analyses. Model 1 examined the associations of psychological distress with the built environment variables with adjustment for individual level covariates. Model 2 examined the association of psychological distress with area level variables (WIMD, slope variability and greenness) also adjusting for individual level covariates. Model 3 examined the association of psychological distress with both built environment and area level variables after adjusting for covariates. The analysis was repeated excluding land use mix in order to investigate the impact of collinearity between land use configuration variables. Statistical analyses were performed with the user-written *runmlwin* command from within Stata 11.2 environment [43]. The command fitted multilevel logistic models with the MLwiN v.2.25 package using the Markov Chain Monte Carlo simulation [44].

## Results

Completed GHQ scores were assessed for 814 men; 745 of who remain within the geographical boundary of the study area, the rest having migrated. After exclusions for missing data across all categories, the study constituted 687 valid responses residing within 34 LSOAs. The number of respondents per LSOA ranged from 6–47, with a mean of 20.2 (SD = 12.7).

The prevalence rate of psychological distress was 19.4% and the mean age of the sample was 73.5 years (SD = 4.3) (Table 1). Mental health was associated with educational attainment, angina, myocardial infarction, high blood pressure and stroke.

**Table 1:** Characteristics of the study sample according to GHQ status

	**Prevalence of psychological distress**		
**Individual level confounders**	**Case (n = 133) GHQ-30 ≥ 5**	**No case (n = 554) GHQ-30 < 5**	**p-value of difference**
Age; Mean (SD)	73.7 (4.3)	73.5 (4.3)	0.549
Alcohol consumption; Mean (SD)	49.8 (100.6)	57.5 (96.7)	0.018
Social class (partly skilled/unskilled); N(%)	25 (18.8)	72 (13.0)	0.085
Educational attainment (None/apprenticeship); N(%)	92 (69.2)	336 (60.6)	0.050
Prevalence of chronic disease			
Heart attack/coronary thrombosis; N(%)	29 (21.8)	70 (12.6)	0.007
Angina; N(%)	46 (34.6)	105 (19.0)	0.005
High blood pressure; N(%)	72 (54.1)	241 (43.5)	0.027
High cholesterol; N(%)	42 (31.6)	163 (29.4)	0.626
Diabetes; N(%)	22 (16.5)	73 (13.2)	0.313
Stroke; N(%)	23 (17.3)	52 (9.4)	0.006

The descriptive statistics of environmental variables within the 1 km street buffer employed in the study have been categorized as dwelling level, land use configuration, topological accessibility of street, natural environment and area level deprivation variables (Table 2). The built environment of Caerphilly ranges from an urban centre to predominantly rural periphery resulting in considerable heterogeneity in the environmental variables.

**Table 2:** Summary of environment variables

**Environmental variables**	**Mean (SD)**	**Range**
**Built environment morphometrics**		
***Dwelling level variables***^***a***^		
Dwelling centred density (within 30 m radius)	14.29 (6.64)	1 – 40
Dwelling types; N (%) Detached	156 (22.70)	
Semi- detached	299 (43.52)	
Terraced	176 (25.63)	
Flats	56 (8.15)	
Plot exposure; N (%) No building fonts exposed to public space	76 (11.06)	
One building fonts exposed to public space	471 (68.56)	
More than one building fonts exposed to public space	140 (20.38)	
***Land use configuration*** (within 1 km street network buffer)^b^		
Land use mix	0.14 (0.03)	0.03 – 0.21
Density of bus stops	23.96 (6.00)	7.66 – 50.46
Density of retail	26.92 (27.58)	1.40 – 140.7
Density of community services	14.36 (9.93)	1.00 – 50.95
Density of recreation & leisure facilities	12.91 (5.11)	2.12 – 34.49
Density of business & offices	30.69 (32.76)	0.87 – 130.53
***Physical accessibility of streets*** (measured for each street segment at varying radii)^c^		
Street movement potential R1200 m	2.40 (0.17)	1.76 – 2.82
Street movement potential R3000 m	3.01 (0.21)	2.40 – 3.54
Street movement potential RNm (all networks segments)	3.63 (0.20)	2.92 – 4.29
Connectivity	3.13 (0.19)	2.66 – 3.65
**Natural environment**		
***Topography*** (variability of slope within 1 km street network buffer)^d^		
Neighbourhood slope variability (*mean)*	3.31	0.99 – 8.04
***Greenness*** (within 500 m airline buffer of dwelling)^e^		
Mean NDVI	0.091	−0.06 – 0.33
**Area-level deprivation**		
WIMD −2005 (within census defined lower super output areas)^f^		
Income domain	24.05 (19.78)	0.34 – 90.32
Employment domain	25.73 (19.14)	1.00 – 95.02
Health domain	25.64 (15.71)	1.70 – 72.3
Education domain	25.82 (16.95)	0.59 – 62.32
Housing domain	10.91 (7.44)	0.19 – 27.23
Physical domain	19.11 (14.63)	1.61 – 65.4

^a^Dwelling level data extracted from Ordnance Survey Address Layer 2.

^b^Land use data was extracted from Ordnance Survey Address Layer 2.

^c^ Street network was extracted from Ordnance Survey Integrated Transport Network Layer, transformed and subjected to space syntax analysis in Confeego. Measured for each street segment at varying radii of capture the effect of multiple urban scales: regional/city scale (N, 3000 m), and local/walking distance scale (1200 m). Thereafter, connectivity and street movement potential were aggregated at the level of a 1 km street network buffer.

^d^Slope raster was processed from the BlueSky 5 metre resolution DTM). Measured as the standard deviation of slope in degrees within 1 km street network buffer.

^e^Greenness was measured as the Normalized Difference Vegetation Index (NDVI) from the 30 m Landsat data.

^f^Area-level deprivation was expressed in terms of the six domain indices of the Welsh Index of Multiple Deprivation, aggregated at the small area level of LSOAs.

A logistic mixed model analysis to assess the associations of psychological distress with measurements of built environment configuration was conducted (Table 3). In all of these analyses (models 1–3) adjustment was made for individual covariates. Analysis of the morphometric variables without adjustment for area level variables (model 1) found that greater local level topological ‘betweenness’ accessibility (*street movement potential* at 1200 metres radius) was associated with lower odds of psychological distress (OR = 0.56, p = 0.02). However, at the regional-level, accessibility captured by global *movement potential* (measured for the entire urban network) was associated with higher risks of psychological distress (OR = 1.53, p = 0.02).

**Table 3:** Two-level logistic mixed effects models with LSOA-level random effects for psychological distress measured by GHQ-30

**Model predictors**	**Model 1**^**†**^	**Model 2**^**†**^	**Model 3**^**†**^
	**O.R. (95% C.I.) p-value**	**O.R. (95% C.I.) p-value**	**O.R. (95% C.I.) p-value**
**Built environment morphometrics**			
***Dwelling level variables***			
Dwelling centred density	1.02 (−0.02, 0.06) p = 0.20		1.01 (−0.03, 0.05) p = 0.32
Plot exposure (none vs. one bldg face)	0.92 (−0.78, 0.57) p = 0.40		0.94 (−0.78, 0.61) p = 0.43
Plot exposure (more than one faces vs. one bldg face)	0.78 (−0.79, 0.27) p = 0.18		0.79 (−0.81, 0.30) p = 0.20
Dwelling type (semi-detached vs. detached)	0.72 (−0.89, 0.23) p = 0.12		0.76 (−0.86, 0.30) p = 0.18
Dwelling type (terraced vs. detached)	0.55 (−1.35, 0.15) p = 0.06*		0.48 (−1.51, 0.02) p = 0.03**
Dwelling type (flat vs. detached)	0.72 (−1.34, 0.64) p = 0.25		0.82 (−1.19, 0.79) p = 0.35
***Land use configuration***			
Land use mix (z-score)			
T2 vs. T1	0.72 (−0.93, 0.27) p = 0.14		0.63 (−1.10, 0.18) p = 0.08*
T3 vs. T1	0.51 (−1.51, 0.19) p = 0.06*		0.42 (−1.77, 0.04) p = 0.03**
Density of bus stops	1.04 (−0.01, 0.10) p = 0.07*		1.04 (−0.02, 0.10) p = 0.07*
Density of retail	0.99 (−0.04, 0.02) p = 0.31		1.00 (−0.04, 0.03) p = 0.45
Density of community services	1.01 (−0.04, 0.06) p = 0.42		1.00 (−0.06, 0.06) p = 0.47
Density of recreation & leisure facilities	0.98 (−0.08, 0.04) p = 0.24		0.98 (−0.08, 0.05) p = 0.33
Density of business & offices	1.02 (0.00, 0.03) p = 0.06*		1.02 (−0.01, 0.04) p = 0.08*
***Topological accessibility of streets (z-score)***			
Street movement potential R1200 m	0.56 (−1.13, -0.01) p = 0.02**		0.54 (−1.28, 0.03) p = 0.03**
Street movement potential R3000 m	0.95 (−0.61, 0.47) p = 0.43		1.14 (−0.69, 0.94) p = 0.38
Street movement potential RN m	1.53 (0.04, 0.81) p = 0.02**		1.24 (−0.39, 0.83) p = 0.25
Connectivity	1.10 (−0.20, 0.39) p = 0.25		1.18 (−0.17, 0.49) p = 0.16
***Natural environment***			
Topography (Standard deviation in slope)		1.24 (−0.03, 0.47)p = 0.04**	1.38 (−0.07, 0.71) p = 0.05**
Greenness (Mean NDVI within 500 m)		0.82 (−0.51, 0.10) p = 0.10*	0.79 (−0.66, 0.21) p = 0.14
***Neighbourhood deprivation***			
WIMD domains			
Income deprivation		1.03 (0.00, 0.07) p = 0.04**	1.03 (−0.02, 0.07) p = 0.11
Employment deprivation		0.97 (−0.07, 0.00) p = 0.03**	0.96 (−0.08, 0.00) p = 0.02**
Health deprivation		0.99 (−0.03, 0.01) p = 0.13	0.99 (−0.03, 0.02) p = 0.31
Education deprivation		1.00 (−0.04, 0.03) p = 0.42	1.02 (−0.03, 0.06) p = 0.21
Housing deprivation		1.00 (−0.04, 0.04) p = 0.47	1.00 (−0.05, 0.04) p = 0.46
Physical environment		1.02 (0.00, 0.04) p = 0.01**	1.02 (0.00, 0.04) p = 0.04**
***Random effects***			
Between LSOA variance (S.E.)	0.054 (0.083)	0.025 (0.039)	0.042 (0.079)
***Model fit***			
Bayesian DIC	690.02	675.53	695.05

Results are expressed as odds ratio, 95% confidence interval and p-value for the logistic regression. All models have been adjusted for individual level variables of age, alcohol consumption, social class, education and prevalence of chronic vascular morbidities.

*T* Tertile (T1, T2, T3 represents the lower, middle and upper tertiles respectively).

*p < 0.10; **p < 0.05.

^**†**^Model 1 comprises of built environmental morphometrics; Model 2 included neighbourhood deprivation captured by six domains of Welsh index of multiple deprivation and natural environment captured by standard deviation in slope and mean greenness index NDVI; Model 3 indicates the fully adjusted model.

Analysis of the area-level variables, without adjustment for morphometric variables (model 2) found that slope variability was associated with higher odds of psychological distress (OR = 1.24, p = 0.04). Greenness measured by the NDVI index was only mildly associated with lower risks of psychological distress (OR = 0.82, p = 0.10). In terms of the WIMD domains, greater income deprivation was associated with higher odds of psychological distress (OR = 1.03, p = 0.04) whilst greater employment deprivation was associated with lower odds of psychological distress (OR = 0.97, p = 0.03). Greater physical environment deprivation was associated with increased risks of psychological distress (OR = 1.02, p = 0.01).

Our fully controlled model with both the morphometric and area level variables together (model 3) found that respondents living in terraced houses exhibited lower odds of psychological distress relative to those living in detached houses (OR = 0.48. p = 0.03). Land use mix was significantly associated with lower odds of psychological distress (OR = 0.42; p = 0.03 for the highest tertile and OR = 0.63; p = 0.08 for the middle tertile). The density of retail or community services or recreation facilities was not related to psychological distress. However, bus stop density (OR = 1.04; p = 0. 07), and density of business and offices (OR = 1.02; p = 0.08) were mildly associated with higher odds of psychological distress in our fully adjusted model. The association of local-level street accessibility (access to opportunities) with lower odds of psychological distress was confirmed (OR = 0.54, p = 0.03) as was the association of slope variability with enhanced risks of psychological distress (OR = 1.38, p = 0.05) whilst, the association of mental health with regional-level street movement potential (a surrogate for traffic-related congestion) was lost. Amongst the deprivation domains, employment deprivation had a slight protective effect (OR = 0.96, p = 0.02) whilst physical deprivation had a slight harmful effect (p = 1,02, p = 0.04). The area level between-LSOA random effects component of the analysis remained small and non-significant throughout our models $\left(\sigma_{u}^{2}<0.10\right)$ indicating negligible between-areas heterogeneity in mental health.

## Discussion

In a sample of community-dwelling older men, and using multi-level methods, we have found that psychological health is associated with both the built environment and area level characteristics. Among the built environment variables, living in areas with terraced housing, greater land use mix and local-level street accessibility (access to destination opportunities at a walking level) were all associated with lower risks of psychological distress. At the area level, higher odds of psychological distress were associated with living in areas with more slope variability and greater income deprivation and lower employment deprivation.

### Strengths and limitations

Strengths of these data lie in the population sample, the detailed assessment of the built environment and the multilevel modeling of putative effects. CaPS has achieved high levels of follow-up throughout and care has been taken to achieve high levels of case ascertainment. The sample used for this analysis represents a 79% response rate in the most recent (5th) phase. Although CaPS was considered sufficiently large at conception the number of men available for the analysis was only just sufficient.

Several previous studies have used composite built environment indices to study their impact upon physical activity [45-48] and mental health [11,12] but do not isolate the effects of specific attributes of built environment upon health. Our study employed a wide range of objectively measured robust and specific spatial metrics of land-use and street-network configurations enabling investigation of their independent effects upon psychological health. Spatial network analysis models of accessibility enable the inclusion of configurational measure of street movement potential which acts as proxies for accessibility in terms of walkability and social connectivity. Within the space syntax paradigm, ‘urban space’, comprising the interconnected sub-spaces and those which are directly linked through better physical connections and longer lines of visual sight, have better systemic accessibility and hence, are associated with higher movement densities of different types (through movement density or destination-seeking movement density). Street configurational measurements of accessibility can thus directly be appended to each street segment, thereby enabling the study of potential independent associations between individual health behaviour and street configuration metric. Another inherent advantage of this technique stems from the fact that it enables the analysis of street segments at varying spatial scales, measuring the impact of accessibility at multiple urban scales - local, community and regional levels. In our models, the through-movement-potential of a street segment indicates the odds of it being used as a through-route while people move through the urban matrix. These measures compliment information over and above more established street connectivity [33,49,50] as well as the GIS-constructed land-use measures of mix and density used in this and other studies. In addition, we used objectively measured natural environment descriptors comprising slope variability and the NDVI index of greenery.

The cross-sectional design limits the confidence with which causal interpretations can be made. The sustained effects of built environment factors upon psychological health through different stages of the life course require further exploration. This can only be achieved through longitudinal analyses. Although adjustment for a wide range of confounders enabled several independent effects of the built environment to be identified, the possibility of residual confounding cannot be excluded. Spatial data were collected as close as possible to the end of CaPS phase V (conducted over the duration of 2002–2004) in order to avoid temporal mismatch [51]. Limitations owing to this are likely to be negligible as data from Caerphilly County Borough Council indicate relatively stabilized land use and street network characteristics.

### Interpretation

Of the dwelling level variables, that terraced housing was positively associated with mental health, and that no other associations were found, suggests a social capital mechanism. For older men, being in closer proximity to neighbours in a walkable neighbourhood may confer social and practical benefits via enhanced degree of acquaintanceship, community ties and neighbourhood satisfaction [26,52,53].

The general hypothesis that greater land use mix is synonymous with greater accessibility to health promoting capital was supported. This is consistent with previous work showing a positive association between land-use mix and neighbourhood satisfaction [13] and between land use mix and physical activity [33,49,50]. However, it does not confirm previous findings from Saarloos et al. who found depression to be positively associated with greater land use mix [15]. Saarloos, acknowledges his findings require further explanation. More specific support for the land use hypothesis in terms of the density of land use types was less clear in our results as statistically significant associations between density of retail, community services, and leisure and recreation units and mental health were not found. However, the study captured the inhibitory effects of specific land uses. The suggestion that density of businesses and offices may be associated with higher odds of psychological distress is consistent with our hypothesis. Areas with predominantly commercial/industrial outlook are usually associated with reduced sense of community; typically associated with unwanted social contacts, having large parking spaces and high traffic [54-56]. The suggestion of an inverse association of psychological distress with density of bus stops was not anticipated. Although this may be a chance effect, it may be that the availability of public transport reduces walking behaviour in older men and this has implications for psychological wellbeing. Alternatively, higher density of public transport routes may in this community be a proxy variable for noise pollution as well as social deprivation that was not assessed through the WIMD.

The space syntax metrics in the model captured the configuration and design of urban form at multiple spatial scales [57]. Higher local-level street accessibility (measured as street movement potential within a radius of 1200 m) was associated with lower odds of psychological distress in all models, controlling for all other variables. Higher local-level accessibility of destinations may help create a walkable, well-integrated community with enhanced accessibility to health promoting capital. Higher regional-level street movement potential was associated with increased odds psychological distress. This might have been attributed to living in the vicinity of major roads, generally associated with traffic density, pollution and reduced perception of safety from traffic, especially among older adults. However, this association was attenuated; no longer being significant in our fully controlled model after the introduction of natural environment and area-level deprivation variables. This was indicative of the moderating effects of natural environment. The slope variability factor introduces the terrain-induced disincentive to going out for longer trips, especially in older adults. Furthermore, the beneficial effect of natural greenness in diluting traffic-related polluting and stress is well known. These findings underscore the positive influence of community-level street-network accessibility upon psychological health and the need for optimizing urban design in this respect.

Among the natural environment variables, slope variability was associated with psychological distress irrespective of the built environment. This may be attributed to reduced physical capacity and mobility in a hilly terrain. A potential beneficial impact of greenness was also found to be independent of the built environment; however our study was underpowered to allow confident interpretation.

CaPS is a cohort of middle-aged and older men; hence, one must be cautious in generalizing the nature of these associations to other sections of the population, particularly on account of differences in individual risk factor profiles between populations e.g. in older women or in younger men. Nevertheless CaPS may be considered a reasonably representative population sample and the data are highly informative for this demographic group.

It is important to recognize that the influence of different components of land use configuration on mental health is likely to be complex and may vary not only according to mix and density but also to the natural and economic environment. For example, CaPS is situated in the South Wales valleys where there is high slope variability and an ongoing post-industrial transition of land use. Under such circumstances it is important to consider the level of generalisability of the observed associations. It may be, for example, that the benefit to mental health of higher local area street accessibility is more widely generalisable than the inverse association of bus-stop density with mental health. Only further research can address these issues. At a more general level, the planning implication of this study lies in the evidence presented that is consistent with the hypotheses that (a) higher land-use mix is associated with enhanced accessibility to health promoting capital and (b) accessibility to destination opportunities by foot is associated with better mental health.

## Conclusions

We have shown that detailed and objective morphometric assessment of the built environment can be applied to an epidemiologic cohort; is informative for health related outcomes; and that the level of detail available has the potential to deliver associations relevant to specific demographic groups within a population. The built environment morphology was assessed both in terms of configuration of land uses, expressed as mix and density of health-specific destinations as well as the configuration of street network assessed through space syntax based network model. Such granularity of evidence will be increasingly important to inform holistic and integrated intervention strategies in preventive public health planning, such as urban design and retro-fitting of the built environment in pursuit of health-sustaining communities. Healthy activity behaviour may be promoted by optimising the distribution and siting of the health-promoting community resources in the urban neighbourhood so as to make them more accessible to the dwelling locations. Similarly, street segments associated with high local movement potential and hence, a higher potential to support pedestrian density may be specifically targeted for further improvements with an aim to enhance neighbourhood walkability thereby promoting walking and social connectivity. In this regard, retrofitting with improved design features and pedestrian infrastructures including nature and quality of sidewalks, proportion of dwelling block faces exposed to sidewalks, presence of traffic calming features and speed impediments as well as enhancements in aesthetic aspects may have positive effects upon healthy activity and behaviour. In relation to older men, our evidence suggests that psychological health benefits from terraced housing (closer proximity between dwellings), greater land use mix, and greater street level accessibility at the local level. Further work is required to explore the causal mechanisms that are involved.

## Competing interests

The authors declare that they have no competing interests.

## Authors’ contributions

CS conceived the study design, conducted the data analysis and prepared the draft of the manuscript. JG and CW provided conceptual and methodological guidance on design and interpretation of results. All the authors participated in revising the manuscript critically for intellectual content. All authors have read and approved the final version of the manuscript.

## Pre-publication history

The pre-publication history for this paper can be accessed here:

http://www.biomedcentral.com/1471-2458/13/695/prepub
